# Characterization of proteogenomic signatures of differentiation of CD4^+^ T cell subsets

**DOI:** 10.1093/dnares/dsac054

**Published:** 2022-12-29

**Authors:** Toshio Kanno, Ryo Konno, Keisuke Miyako, Takahiro Nakajima, Satoru Yokoyama, Shigemi Sasamoto, Hikari K Asou, Junichiro Ohzeki, Yusuke Kawashima, Yoshinori Hasegawa, Osamu Ohara, Yusuke Endo

**Affiliations:** Department of Frontier Research and Development, Laboratory of Medical Omics Research, Kazusa DNA Research Institute, Kisarazu, Chiba 292-0818, Japan; Department of Applied Genomics Kazusa DNA Research Institute, Kisarazu, Chiba 292-0818, Japan; Department of Applied Genomics Kazusa DNA Research Institute, Kisarazu, Chiba 292-0818, Japan; Department of Frontier Research and Development, Laboratory of Medical Omics Research, Kazusa DNA Research Institute, Kisarazu, Chiba 292-0818, Japan; Department of Frontier Research and Development, Laboratory of Medical Omics Research, Kazusa DNA Research Institute, Kisarazu, Chiba 292-0818, Japan; Department of Frontier Research and Development, Laboratory of Medical Omics Research, Kazusa DNA Research Institute, Kisarazu, Chiba 292-0818, Japan; Department of Frontier Research and Development, Laboratory of Medical Omics Research, Kazusa DNA Research Institute, Kisarazu, Chiba 292-0818, Japan; Department of Frontier Research and Development, Laboratory of Medical Omics Research, Kazusa DNA Research Institute, Kisarazu, Chiba 292-0818, Japan; Department of Applied Genomics Kazusa DNA Research Institute, Kisarazu, Chiba 292-0818, Japan; Department of Applied Genomics Kazusa DNA Research Institute, Kisarazu, Chiba 292-0818, Japan; Department of Applied Genomics Kazusa DNA Research Institute, Kisarazu, Chiba 292-0818, Japan; Department of Frontier Research and Development, Laboratory of Medical Omics Research, Kazusa DNA Research Institute, Kisarazu, Chiba 292-0818, Japan; Department of Omics Medicine, Graduate School of Medicine, Chiba University, Chiba 260-8670, Japan

**Keywords:** immunology, CD4 T cell, proteogenomics, RNA sequencing, liquid chromatography-assisted mass spectrometry

## Abstract

Functionally distinct CD4^+^ helper T (Th) cell subsets, including Th1, Th2, Th17, and regulatory T cells (Treg), play a pivotal role in the regulation of acquired immunity. Although the key proteins involved in the regulation of Th cell differentiation have already been identified how the proteogenomic landscape changes during the Th cell activation remains unclear. To address this issue, we characterized proteogenomic signatures of differentiation to each Th cell subsets by RNA sequencing and liquid chromatography-assisted mass spectrometry, which enabled us to simultaneously quantify more than 10,000 protein-coding transcripts and 8,000 proteins in a single-shot. The results indicated that T cell receptor activation affected almost half of the transcript and protein levels in a low correlative and gene-specific manner, and specific cytokine treatments modified the transcript and protein profiles in a manner specific to each Th cell subsets: Th17 and Tregs particularly exhibited unique proteogenomic signatures compared to other Th cell subsets. Interestingly, the in-depth proteome data revealed that mRNA profiles alone were not enough to delineate functional changes during Th cell activation, suggesting that the proteogenomic dataset obtained in this study serves as a unique and indispensable data resource for understanding the comprehensive molecular mechanisms underlying effector Th cell differentiation.

## 1. Introduction

After antigenic stimulation through the T cell receptor (TCR), quiescent naïve CD4^+^ T cells undergo clonal expansion and acquire an effector phenotype.^1,2^ In addition to TCR-mediated signal transduction, environmental cytokines are also essential commitment factors for instructing the differentiation of T cells into effector and regulatory cell types.^1,2^ In particular, naïve CD4^+^ T cells can differentiate following induction by lineage-specifying cytokines into functionally distinct subsets, including Th1, Th2, Th17, and regulatory T cells (Tregs).^1,2^ IFNγ-producing Th1 cells are essential for obtaining immunity to intracellular pathogens, and IL-4-producing Th2 cells promote immunity to parasite infection as well as allergic inflammation.^1–3^ IL-17-producing Th17 cells are implicated in protection against fungal pathogens as well as many types of autoimmune disorders.^1,2,4^ Tregs are critical for immunological tolerance and immune homeostasis.^1,2,5^ The Th17/Treg balance is crucial for preventing excessive immune responses, autoimmune disorders, and metabolic-syndrome pathogenesis.^1,2^ Notably, the expression of lineage-specific transcription factors is required for Th cell differentiation. For example, T-bet (encoded by *Tbx21*) and GATA3 are lineage-determining regulators for Th1 and Th2 cells.^1,2^ Similarly, as Foxp3 is to Tregs, RORγt is essential for the Th17 cell differentiation and function.^1,2,4,5^ The appropriate expression of these transcription factors and the effector cytokines they control is therefore critical for proper immunoregulation.

The differentiation of Th cell subsets is accompanied by dynamic changes in the phenotype, as revealed by genome-wide transcriptomic and epigenome analyses. Although the central dogma in biology describes the flow of genetic information from DNA to RNA to proteins, there is not always a correlative relationship between the concentration of transcripts and proteins.^6^ Systematic studies quantifying transcripts and proteins in a genome-wide manner, conventionally termed ‘proteogenomic analyses’, have revealed the importance of multiple processes after transcription that help control the production of a protein.^7–9^ In fact, it is becoming increasingly apparent that post-transcriptional and post-translational regulation of Th cell responses is required for a better understanding of cellular events that occur during the differentiation of these subsets.^9–11^ Post-transcriptional events are mediated by RNA-binding proteins and/or non-coding RNA that recognize specific cis-regulatory elements on target mRNAs.^11–13^ ZFP36L2, an RNA-binding protein, hampers the recruitment of preformed mRNA to ribosomes and blocks its translation into protein, thus preventing aberrant cytokine production in CD4^+^ T cells in mice.^11^ miR29 suppresses murine CD4^+^ T cell responses by directly targeting mRNA encoding IFNγ or T-bet.^12,13^ In addition, a comprehensive analysis was performed to assess post-translational modifications, including protein phosphorylation and ubiquitination, to reveal activated signalling pathways and protein lifespans.^14,15^ However, due to the lack of genome-wide proteogenomic datasets including both mRNA and protein profiles simultaneously determined from the same cell ensemble, we cannot capture overall changes in mRNA and protein abundance accompanied by Th cell differentiation in a molecular term.

Advances in technology, such as mass spectrometry and the development of next-generation sequencing, have changed our approach to tackling difficult biological questions at a systemic level. Applications of these new technologies have led to the identification of many biomarkers for complex biological phenomena and proteins that regulate pathological conditions, thus facilitating the elucidation of disease mechanisms. In this regard, we developed a proteogenomic analysis using a phenol–guanidinium isothiocyanate (P/GTC) reagent, which allows for the extraction of DNA, RNA, and proteins from the same lysate of a single sample.^16^ P/GTC-based sample preparation can obtain RNA and proteins from the same lysate, thus allowing for a more accurate proteogenomic analysis. Furthermore, recent advances in liquid chromatography-coupled mass spectrometry in data-independent acquisition mode (DIA-LC–MS/MS) have allowed us to more accurately conduct comparative analysis of mRNA and protein profiles in similar depth.^17,18^ Thus, we are ready to carry out simultaneous mRNA and protein profiling in depth, which is enough to be called proteogenomic analyses.

In this study, we assessed the proteogenomic profiles of naïve CD4^+^ T cells to five different cytokine combinations following TCR stimulation, using a combination of our newly developed methods together with other established ones. A previous report showed that the transcriptome alone cannot explain the full extent of biological phenomenon and emphasized the importance of post-transcriptional regulation of proteins.^19^ That study also clarified that the presence of a low correlation between quantitative changes in mRNA and protein levels during Th cell differentiation was the rule rather than an exception. We therefore consider proteogenomic analyses to function as a new framework for obtaining a comprehensive understanding of the dynamic changes in the proteogenomic state during effector Th cell differentiation.

## 2. Materials and method

### 2.1. Mice

C57BL/6 mice were purchased from CLEA Japan. All mice were used at 6–8 weeks old and were maintained under specific-pathogen-free conditions. Almost equal number of male and female animal was used for this study. The animal experiments were performed with protocols approved by the Institution Animal Care and Use Committee of KAZUSA DNA research institute (Registration number: 30-1-002). Experiments and animal care were performed according to the guidelines of Kazusa DNA Research Institute.

### 2.2. Cell preparation

Splenic naïve CD4^+^ T cells were obtained by the negative selection using the Mojo Sort Mouse CD4 T Cell Isolation Kit (Biolegend #480006) and positive selection using CD62L MicroBeads, mouse (Miltenyi Biotec #130-049-701). Naïve CD4^+^ T cells were plated onto 24-well tissue culture plates (Costar #3526) pre-coated with 10 mg/ml anti-TCRβ antibody (H57-597, BioLegend) for 2 days. Culture medium contained 1 μg/ml anti-CD28 antibody (clone 37.51, BioLegend) and cytokine that induce differentiation of Th cell subsets. Th0 cell cultures contained 15 ng/ml IL-2 (WAKO), 1 μg/ml anti-IL-4 antibody (BioLegend) and 1 μg/ml anti-IFNγ antibody (BioLegend). Th1 cell cultures contained 15 ng/ml IL-2, 10 ng/ml recombinant mouse IL-12 (WAKO) and 1 μg/ml anti-IL-4 antibody. Th2 cell cultures contained 15 ng/ml IL-2, recombinant mouse 10 ng/ml IL-4 (WAKO) and 1 μg/ml anti-IFNγ antibody. Th17 cell cultures contained 10 ng/ml IL-6 (BD biosciences), 1 ng/ml TGFβ (BD biosciences), 1 μg/ml anti-IL-2 antibody (BioLegend), 1 μg/ml anti-IL-4 antibody and 1 μg/ml anti-IFNγ antibody. Regulatory T cell cultures contained 30 ng/ml IL-2, 10 ng/ml TGFβ, 1 μg/ml anti-IL-4 antibody and 1 μg/ml anti-IFNγ antibody.

### 2.3. Sample preparation for RNA-seq and proteomics analysis

Th cell subsets were cultured as Cell Preparation. After 2 days cell culture, cells were collected and stained with anti-Annexin V FITC (BioLegend #640906) for 20 min on ice and then incubated with anti-FITC MicroBeads (Miltenyi Biotec #130-048-701) for 20 min on ice. Live Th cell subsets were obtained by the negative selection. The cell pellet was mixed with 0.8 ml of TRIzol reagent (Thermo Fisher Scientific #15596-018) (phenol-guanidinium isothiocyanate reagent: P/GTC reagent) and then was stored at −80°C until use. RNA was isolated from the cell samples using P/GTC reagent according to the manufacturer’s protocol. In brief, the frozen cells mixed with P/GTC reagent were thawed at room temperature and were separated into a clear upper aqueous phase (containing the RNA) by the addition of 0.2 ml of chloroform. RNA was precipitated from the aqueous phase with 0.5 ml of isopropanol. The precipitated RNA was washed and then redissolved for use in downstream processing. Proteins were isolated from the cell samples in P/GTC reagent with a slight modification in the previously reported procedure.^16^ In brief, proteins were precipitated from the phenol/ethanol phase by adding 0.8 ml of ACN, and the samples were incubated at room temperature for 10 min and then were centrifuged at 15, 000 *g* for 15 min at 4°C. The protein pellet was once washed with 0.8 ml of ACN and then was redissolved in 100 mM Tris–HCl (pH 8.5) containing 2% SDS by sonication using Bioruptor II (CosmoBio). Protein concentration in the protein extract was determined using a BCA protein assay kit (Thermo Fisher Scientific # 23225) and adjusted to 1 μg/μl with 100 mM Tris–HCl (pH 8.5) containing 2% SDS. As previously reported,^18^ the 20 µl of protein extract was reduced and alkylated, then digested by trypsin/Lys-C Mix (Promega # V5072), followed by desalting with SDB STAGE tip (GL Sciences Inc. # 7820-11200).

### 2.4. 3ʹ mRNA-seq library preparation

TRIzol reagent was used for the extraction of total cellular RNA and Quantus Fluorometer (Promega #E6150) was used for determining of RNA concentrations. Total 500 ng of RNA was used for the 3ʹ mRNA library preparation with QuantSeq 3ʹ mRNA-Seq Library Prep Kit FWD (LEXOGEN #015.384) according to the manufacture’s protocol. After the PCR step, size distribution and yield of the library was determined by the D1000 high sensitivity tape station (Agilent #5067-5582) or Agilent High Sensitivity DNA kit on the bioanalyzer (Agilent #5067-5583). The pooled libraries were loaded on the Illumina Nextseq500 platform and analysed by 75bp single read.

### 2.5. Analysis of RNA-seq data

Adaptor sequences were trimmed from the raw RNA-seq reads with fastp (v 0.23.1).^20^ Trimmed reads of each sample were mapped to the reference mouse genome mm10 by using STAR (v 2.3.1)^21^ and normalized to 1 million reads in the original library. Genes with an average of 5 or more reads in either group were subjected for further analysis. Two-fold changed genes were defined as differentially expressed genes. PCA analysis and heatmap were depicted with R software (https://cran.r-project.org/) (v 3.6.0).

### 2.6. DIA-LC–MS/MS

About 500 ng of peptides was directly injected onto a 75 μm × 20 cm PicoFrit emitter (New Objective # PF360-75-8-N-5) packed in-house with C18 core–shell particles (CAPCELL CORE MP 2.7 μm, 160 Å material; Osaka Soda # 51227 (disassembled the column and got the particles)) at 50°C and then separated with a 120-min gradient at a flow rate of 100 nl/min using an UltiMate 3000 RSLCnano LC system (Thermo Fisher Scientific). Peptides eluting from the column were analysed on a Q Exactive HF-X (Thermo Fisher Scientific) for overlapping window DIA-MS.^18^ MS1 spectra were collected in the range of 495-745 m/z at 30,000 resolution to set an automatic gain control (AGC) target of 3e6 and maximum injection time of ‘auto’. MS2 spectra were collected in the range of more than 200 m/z at 45,000 resolution to set an AGC target of 3e6, maximum injection time of ‘auto’, and stepped normalized collision energy of 22%, 26%, and 30%. The overlapping window patterns at m/z 500–740 (isolation window width, 4 Da) were used for window placements optimized via Scaffold DIA v2.1.0.

MS files were searched against a mouse spectral library using Scaffold DIA v2.1.0 (Proteome Software, Inc., Portland, OR). The mouse spectral library was generated from the mouse UniProtKB/Swiss-Prot protein sequence database (proteome ID UP000000589, reviewed, canonical, 17,021 entries, downloaded on 29 November 2019) by Prosit.^22,23^ The Scaffold DIA search parameters were as follows: experimental data search enzyme, trypsin; maximum missed cleavage sites, 1; precursor mass tolerance, 8 ppm; fragment mass tolerance, 10 ppm; static modification, cysteine carbamidomethylation. The protein identification threshold was set both peptide and protein false discovery rates of less than 1%. Protein and peptide quantification was calculated by EncyclopeDIA algorithm^24^ in Scaffold DIA v2.1.0.

The MS files have been deposited to the ProteomeXchange Consortium via the jPOST partner repository^25^ with the dataset identifier PXD036065.

### 2.7. Analysis of proteomics data

The value of protein intensity was transformed to log2, and then each protein was filtered to contain more than 70% valid value in at least one group. The remaining missing values were imputed by random numbers drawn from a normal distribution (width, 0.3; downshift, 2.8) in Perseus v1.6.15.0.^26^ 2.0-fold changed genes were defined as differentially expressed proteins. PCA analysis and heatmap were depicted with R software (https://cran.r-project.org/) (v 3.6.0).

### 2.8. Quantitative real-time PCR

Total RNA was isolated with the TRIzol reagent (Invitrogen #15596-018). cDNA was synthesized with an oligo (dT) primer and Superscript II RT (Invitrogen #18064-014). Quantitative RT-PCR was performed using TB Green Real Time PCR kit (Takara #RR820A).^27^ Primers were purchased from Thermo Fisher Scientific. Gene expression was normalized with the Hprt mRNA signal or the 18S ribosomal RNA signal. Primer sequences used in this study are shown below.

*18S*_FW: 5’-AAATCAGTTATGGTTCCTTTGGTC-3’

*18S*_RV: 5’-GCTCTAGAATTACCACAGTTATCCAA-3’

*Hprt*_FW: 5’-TCCTCCTCAGACCGCTTTT-3’

*Hprt*_RV: 5’-CCTGGTTCATCATCGCTAATC-3’

*Foxp3*_FW: 5’-AGAAGCTGGGAGCTATGCAG-3’

*Foxp3*_RV: 5’-ACTGGTGGCTACGATGCAG-3’

*Gata3*_FW: 5’-TTATCAAGCCCAAGCGAAG-3’

*Gata3*_RV: 5’-AGACCGGGTCCCCATTAG-3’

*Irf7*_FW: 5’-CTTCAGCACTTTCTTCCGAGA-3’

*Irf7*_RV: 5’-TGTAGTGTGGTGACCCTTGC-3’

*Rorc*_FW: 5’-ACCTCTTTTCACGGGAGGA-3’

*Rorc*_RV: 5’-TCCCACATCTCCCACATTG-3’

*Tbx21*_FW: 5’-CAACCAGCACCAGACAGAGA-3’

*Tbx21*_RV: 5’-ACAAACATCCTGTAATGGCTTG-3’

*Tnfsf8*_FW: 5’-GCAAAGGACCCTCCAATCCA-3’

*Tnfsf8*_RV: 5’-TCGCACTTGATGACAACCGA-3’

### 2.9. FACS analysis

For surface staining, anti-CD30L PE (1:200, RM153, BD Biosciences #106405), anti-PD-L2 PE (1:200, TY25, Biolegend #107205), and anti-CCR6 BV421 (1:200, 140705, BD Biosciences #564736) was stained for 30 min on ice and dead cell was stained with Propidium iodide (1:2,000, DOJINDO #341-07881) before FACS analysis. For intracellular staining, dead cells were first stained with Fixable Viability Dye eFluor 780 (1:1,000, eBioscience #65-0865-14) for 10 min. For T-bet, GATA3, RORγt, FOXP3, and IRF7 staining, sample preparation was conducted with Lyse/Fix buffer for 10 min at 37°C (BD Biosciences #558049) and Perm buffer III (BD Biosciences #558050) for 30 min on ice according to the manufacture’s protocol. Cells were stained with anti-T-bet PE (1:50, 4B10, BioLegend #644809), anti-GATA3 Alexa488 (1:50, L50-823, BD Biosciences #560163), anti-RORγt BV421 (1:50, Q31-378, BD Biosciences #562894), anti-FOXP3 Alexa647 (1:50, MF23, BD Biosciences #560401), or anti-IRF7 PE (1:200, MNGPKL, eBioscience #12-5829-82) for 45 min in the dark. Flow cytometric data were analysed after removal of dead cells and doublets cells with Flowjo software (version 10.4).

### 2.10. Statistics and reproducibility

Data are expressed as mean ± s.d.. The data were analysed with the Graphpad Prism software program (version 7). Differences were assessed using unpaired two-tailed student *t* tests or one-way ANOVA followed by tukey’s multiple comparisons test. Differences with *P* values of <0.05 were considered to be significant. No data were excluded from the analysis of experiments. Mice were commercially sourced and randomized into experimental groups upon arrival, and all animals within a single experiment were processed at the same time. For RNA-sequencing and proteome analyses, the investigator was blinded. Data display similar variance between groups and are normally distributed where parametric tests are used.

## 3. Results

### 3.1. TCR stimulation largely reprograms the proteogenomic profiles of Th cells

**Figure 1.**
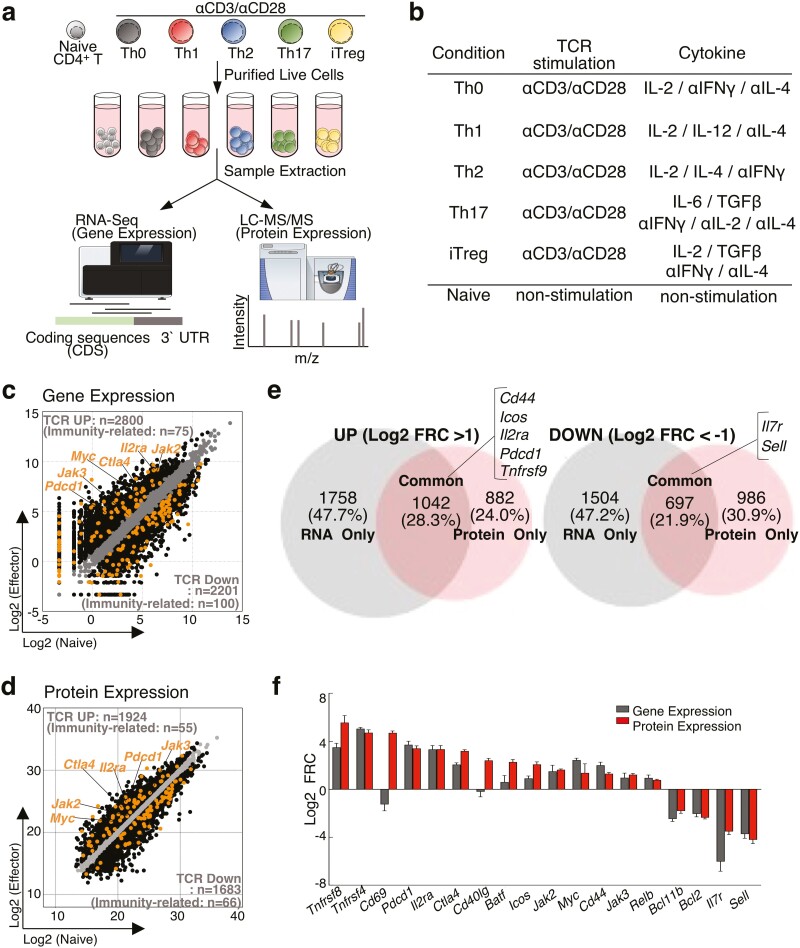
A combination of global RNA-seq and proteome analyses revealed the effects of TCR stimulation on mRNA and protein expression profiles. (a) Overview of the experimental design. (b) List of cytokine conditions. (c and d) A scatter plot of gene or protein expression profiles by RNA-sequencing (c) or proteome analysis (d) compares in naive and Th0 cells. (e) Venn diagram showed overlaps and differences between 2.0-fold increased (Left panel) or decreased (Right panel) genes in naive and Th0 cells. (f) Bar plots showed fold changes of mRNA and protein expression immunity-related genes in Th0 cells relative to naive T cells.

To assess dynamic changes in mRNA and protein levels during T cell differentiation, we performed RNA sequencing and DIA-LC–MS/MS.^17^ As shown in Fig. 1a and b, naïve CD4^+^ T cells were stimulated with immobilized anti-TCR mAb and anti-CD28 mAb for 48 h under Th0, Th1, Th2, Th17, or induced regulatory T cell (iTregs) culture conditions (Fig. 1a and b). Because TCR stimulation caused activation-induced cell death, dead cell removal was performed before sample preparation.^28^ Using these samples, we developed a proteogenomic analysis using a phenol–guanidinium isothiocyanate (P/GTC) reagent, which allows for simultaneous recovery of RNA and proteins from the same lysate of a single sample. Since TCR stimulation-mediated activation is a fundamental step in most T cell responses, we first compared the changes in mRNA and protein levels between activated Th0 and quiescent naïve CD4^+^ T cells. Th0 cells were cultured under non-polarized conditions to eliminate the influence of cytokine-mediated activation (see “Methods” section). In total, we prepared four samples in each group and were able to detect 10,435 protein-coding transcripts and 8,312 proteins from a single lysate (Supplementary Tables 1 and 2). The differentiation signature from naïve CD4^+^ T cells to Th0 cells was thus derived as a proteogenomic signature and divided into three classes: 1,042 genes exhibiting concordant changes at both the mRNA and protein levels (i.e. as a proteogenomic signature); 1,758 genes only changed at the mRNA level; 882 genes only changed at the protein level. Scatter plots show dramatic changes in the mRNA and protein levels during Th cell activation (UP:2800, DOWN:2201 in RNA profile and UP:1924, DOWN:1683 in protein profile) (Fig. 1c and d). We also observed that the expression of immune response-related molecules, including αβT cell differentiation, adaptive immune response and cytokine activity, was also changed at the mRNA and protein levels (Fig. 1c and d and Supplementary Table 2). In particular, well-established T cell activation markers, including *Cd44*, *Icos*, *Il2ra*, *Pdcd1*, and *Tnfrsf9*, were highly upregulated (Fig. 1e and f). Conversely, the levels of *Il7ra* and *Sell*, which are markers of T cell quiescence, were downregulated in activated Th0 cells, as expected (Fig. 1f). Taken together, the mRNA and protein levels of these well-established T cell activation/quiescence markers changed in a well-correlated manner (Fig. 1e and f), except for *Cd69* and *Cd40lg*. Although the discrepancy of changes in Cd69 and Cd40lg at the mRNA and protein levels was apparently exceptional among T cell activation/quiescence markers, it should be noted that a large number of differentially expressed genes exhibited changes either only at the mRNA level or only at the protein level (Fig. 1e and f).

### 3.2. Proteogenomic profiles showed the T cell activation status and Th cell subset signature

Next, to understand the whole differentiation process into each Th cell subset, we compared the proteogenomic profiles between each activated Th cell subset and quiescent naïve CD4^+^ T cells. A principal-component analysis (PCA) illustrated that the large impact on the proteogenomic profiles was due to TCR-mediated activation rather than cytokine stimulation (Supplementary Fig. 1a and b and Supplementary Table 3). We also found that Th17 and iTregs formed a cluster that was distinct from other activated Th cell clusters consisting of Th0, Th1, and Th2 cells. Gene ontology and pathway analyses of the differentially expressed genes of Th cell subsets using the NIAID DAVID and KEGG databases showed common features with all Th cell subsets: significant enrichment of functional categories related to rapid cell proliferation, including Cell cycle, Cell division, and DNA replication for upregulated genes; and the creation of a category of activation of transcription and protein phosphorylation, including *Dgka, Dgkd*, and *TBk1*, for downregulated genes (Supplementary Fig. 1c). Thus, our proteogenomic analyses faithfully clarified the molecular signatures of TCR activation for many aspects of T cell biology, including the cell differentiation, function, and eventual fate, as previously reported.^29^

**Figure 2.**
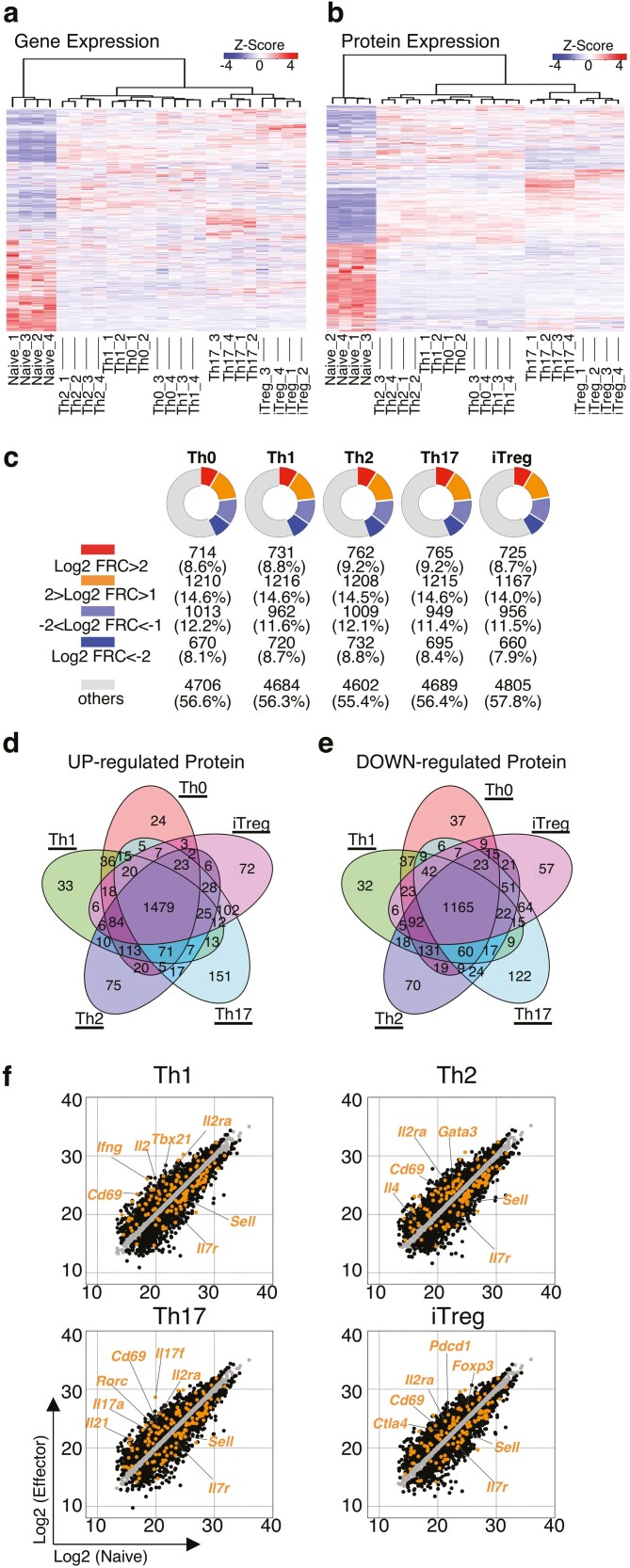
Protein expression analysis showed T cell activation status and Th cell subset signature. (a and b) A clustering heatmap depict the gene (a) or protein expression (b) in naive, Th0, Th1, Th2, Th17, and iTregs cells. (c) Pie chart showed the number of differentially expressed proteins in Th0, Th1, Th2, Th17, or iTregs cells relative to naive T cells (*n* = 4 per genotype). (d and e) Venn diagram showed overlaps and differences between 2.0-fold increased (d) or decreased (e) genes in Th0, Th1, Th2, Th17, or iTregs cells relative to naive T cells. (f) A scatter plot of protein expression profiles by proteome analysis compares in Th1, Th2, Th17, and iTregs cells relative to naive T cells.

Consistent with the PCA, a clustering heat map demonstrated distinct patterns between quiescence naïve CD4^+^ T cells and activated Th cell subsets at both the mRNA and protein levels (Fig. 2a and b). Notably, the expression profile of mRNA and protein in Th17 and iTregs was far different from that in Th0, Th1, and Th2 cells (Fig. 2a and b). TGFβ is a commonly used cytokine to induce both Th17 and iTreg cell differentiation, suggesting that TGFβ-mediated signalling may contribute to the profiles in gene and protein expression of these subsets.^30,31^ Although TCR stimulation changed a large number of genes at the mRNA level, more than half of differentially expressed genes exhibited a change only at the mRNA level as shown in Fig. 1e and f. We, therefore, next focussed on proteins that were differentially expressed compared to naïve CD4^+^ T cells in order to understand the effects of TCR stimulation. A deeper analysis showed that the number of upregulated proteins (Log2 FRC>1) was 1924, 1947, 1970, 1980, and 1892 in Th0, Th1, Th2, Th17, and iTregs, respectively (Fig. 2c). We also observed that the number of downregulated proteins (Log2 FRC<-1) was 1683, 1682, 1741, 1644, and 1616 in Th0, Th1, Th2, Th17, and iTregs, respectively (Fig. 2c). Each Th cell subset shared most of the differentially expressed proteins in common (Fig. 2d and e) (UP:1479, DOWN:1165), probably due to TCR activation. In fact, the list of the differentially modulated genes included highly upregulated T cell activation markers, such as *Il2ra* and *Cd69*, and downregulated marker proteins for resting T cells, such as *Il7r* and *Sell* (Fig. 2f and Supplementary Table 4). Several transcription factors that control the differentiation and function of these Th cell subsets as well as the expression of master transcription factors specific to each Th cell subset,^1,2^ including T-bet, GATA3, RORγt, and FOXP3, were highly upregulated in Th1, Th2, Th17, and iTregs, respectively (Fig. 2f). Consistent with the upregulation of master transcription factors, we observed elevated levels of signature cytokines, including IFNγ, IL-4, and IL-17A, in each Th cell subset. These data showed that the proteomic analysis reflects the Th cell activation status and lineage-specific signature in each Th cell subset.

### 3.3. The differential proteogenomic signatures characteristic to each Th cell subsets were determined

**Figure 3.**
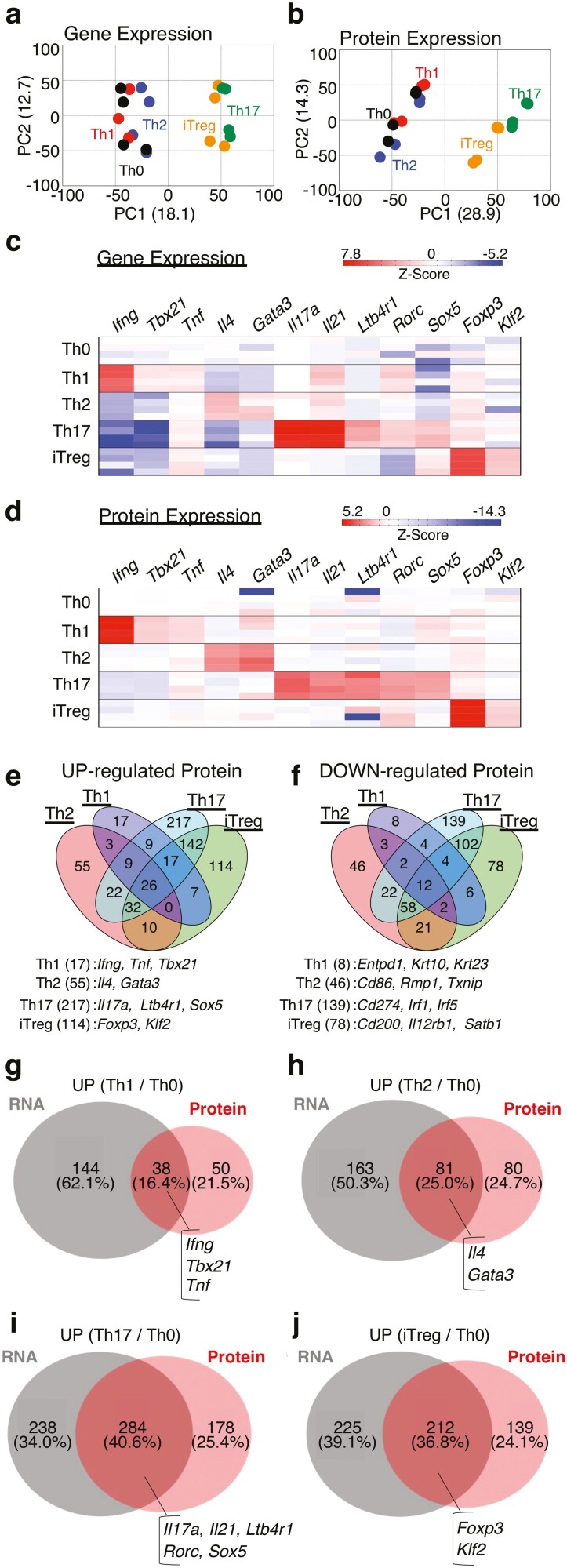
Cytokine responses caused the differences in RNA and protein expression in Th cell subsets compared to Th0 cells. (a and b) PCA plot of gene or protein expression profiles by RNA-sequencing (a) or proteome analysis (b), including Th0, Th1, Th2, Th17, and iTregs cells. (c and d) heat map depicts gene (c) or protein (d) expression of the T cell subset specific signature (*n* = 4 per genotype). (e and f) Venn diagram showed overlaps and differences between 2.0-fold increased (e) or decreased (f) proteins in Th1, Th2, Th17, or iTregs cells relative to Th0 cells. Genes or protein varied in each subset alone are listed at the bottom. (g–j) Venn diagram showed overlaps and differences between 2.0-fold increased genes in Th1 (g), Th2 (h), Th17 (i), and iTregs cells (j) as compared to Th0 cells.

We next characterized differential proteogenomic signatures of Th cell subsets by comparing the proteogenomic profiles of Th1, Th2, Th17, and iTregs to that of Th0 cells. Consistent with the clustering heat map (Fig. 2a and b), PCAs shows that the proteogenomic profiles of Th17 and iTregs were closer to each other than those of the remaining Th cell subsets, Th1, Th2, and Th0, as shown in Fig. 3a and b (Supplementary Table 5). We also confirmed that subset-specific cytokine and receptor mRNAs and proteins were upregulated concordantly (Th1: *Ifng,* and *Tnf*, Th2: *Il4*, Th17: *Il17a*, *Il21*, and *Ltb4r1*) (Fig. 3c and d). A similar tendency was observed in subset-specific transcription factors (Th1: *Tbx21*, Th2: *Gata3*, Th17: *Rorc* and *Sox5*, iTregs: *Foxp3* and *Klf2*) (Fig. 3c and d). Although the *Il17a, Sox5*, and *Foxp3* mRNA expression was also upregulated two-fold in Th cell subsets except Th17 and iTregs (Supplementary Fig. 2a), the protein expression of *Il17a, Sox5*, and *Foxp3* was upregulated more than two-fold only in Th17 and iTreg cells compared to control Th cells (Fig. 3e). Thus, we next focussed on the protein expression profile to assess how many genes are selectively modulated in each Th cell subset compared to Th0 cells. The proteomic analysis revealed that the number of proteins that were altered in specific Th cell subsets varied among subsets (Fig. 3e and f). Th1 cells had the lowest number of differentially expressed subset-specific proteins compared to Th0 cells (UP: 17 proteins, DOWN: 8 proteins). Th2 cells had a moderate number of changed proteins relative to Th0 cells (UP: 55 proteins, DOWN: 46 proteins). Consistent with PCA results, the protein expression profiles of Th17 cells and iTregs were markedly changed compared to Th0 cells (for Th17, UP: 217 proteins, DOWN: 139 proteins; for iTregs, UP: 114 proteins, DOWN: 78 proteins). We also observed that lineage-specific signatures, including cytokines and transcription factors, were selectively upregulated in each Th cell subset (Fig. 3e). Furthermore, the selective downregulation of immune response-related proteins in Th cell subsets was detected (Fig. 3f and Supplementary Fig. 2b, Th1: *Entpd1*, Th2: *Cd86*, Th17: *Cd274, Irf1*, and *Irf5*, iTreg: *Cd200, Il12rb1,* and *Satb1*). Thus, most genes that determine the Th cell subset differentiation were found to be markedly changed at both the mRNA and protein levels.

However, similar to our findings for TCR-stimulated activation (Fig. 1e), most subset-specific genes showed changes only at the mRNA or protein level (Fig. 3g–j). In Th1 cells, most genes that were increased at the mRNA level did not show differences in the expression at the protein level (RNA-only; 144 genes, common: 38 genes). In contrast to Th1 cells, Th2 cells had a moderate number of differentially upregulated genes at both the mRNA and protein levels (RNA-only: 163 genes, common: 81 genes). Th17 and iTregs showed changes at the mRNA and protein levels in almost half of the differentially expressed genes (for Th17, RNA-only: 238 genes, common: 284 genes; for iTregs, RNA-only: 225 genes, common: 212 genes). Regarding downregulated genes, the same tendency was observed in each Th subset compared to control Th0 cells (Supplementary Fig. 2c–f). Taken together, these findings indicate that although we observed strong upregulation of subset-specific genes at both the mRNA and protein levels, more than half of differentially expressed genes showed an uncorrelated expression of RNA and protein in Th cell subsets.

### 3.4. The differential modulation between the mRNA and protein levels during Th cell differentiation was confirmed

**Figure 4.**
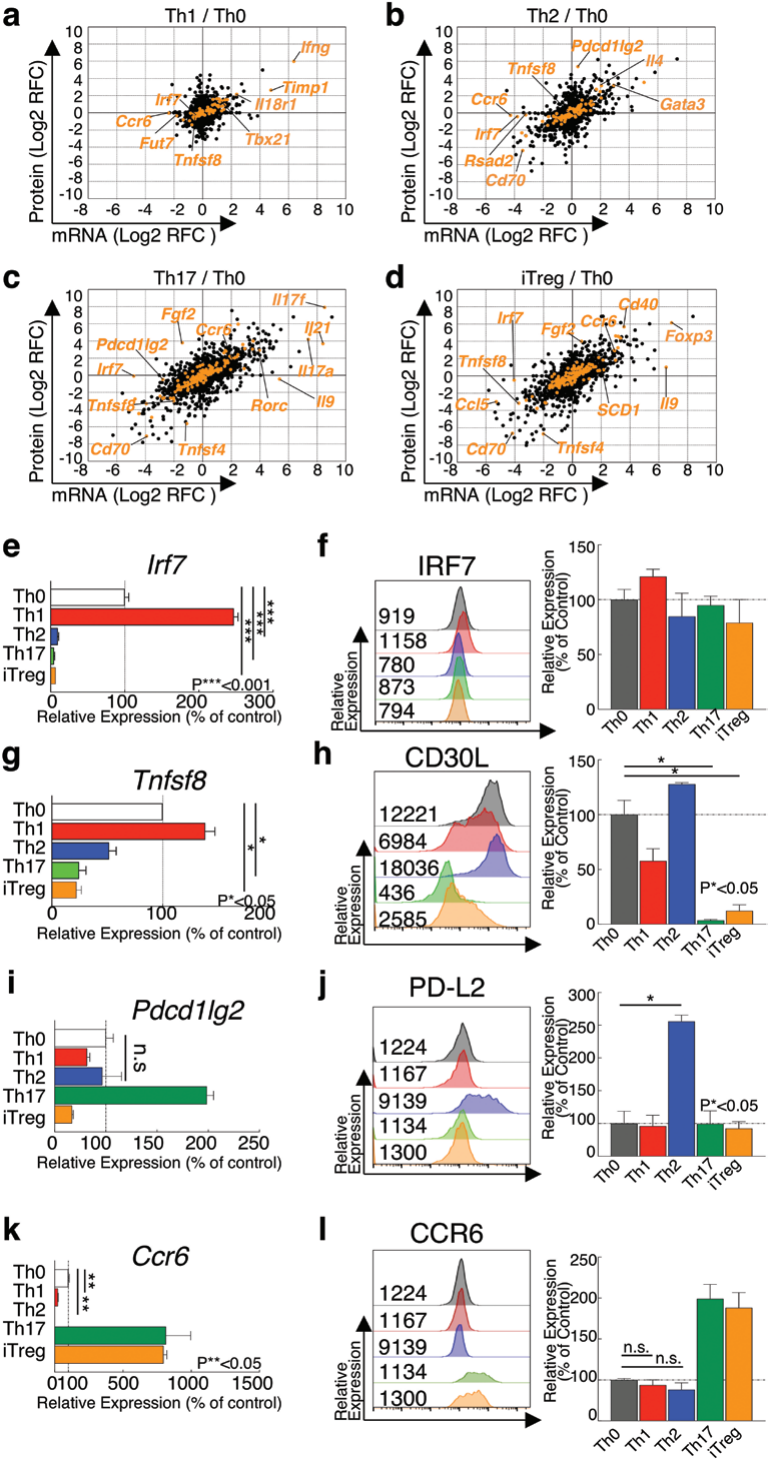
Cytokine stimulation caused uncorrelated expression of RNA and Protein in Th cell subsets. (a–d) A scatter plot of gene and protein expression profiles compares in Th1 (a), Th2 (b), Th17 (c), or iTregs cells (d) to Th0 cells. (e, g, i, and k) qRT-PCR analyses of the relative expression of *Irf7* (e), *Tnfsf8* (g), *Pdcd1lg2* (i), or *Ccr6* (k) in Th1, Th2, Th17, or iTregs cell compared to Th0 cells. Relative expression (normalized to *Hprt*) with s.d. is shown. (f, h, j, and l) Intracellular staining or surface staining of flow cytometry analysing of IRF7 (f), CD30L (h), PD-L2 (j), or CCR6 (l) in Th0, Th1, Th2, Th17, or iTregs cell. Mean fluorescence intensity (MFI) is shown. Summary data of three independent experiments of each protein expression are shown here. Data are means ± s.d. (*n* = 3 per each group biologically independent sample).

The combined transcriptome and proteome analyses revealed that most of these subset-specific cytokines and transcription factors increased by more than 2-fold at both the mRNA and protein levels (Fig. 4a and d). Furthermore, our combined analyses also showed that some unique proteins were uncorrelated with the mRNA levels in each Th cell subset (Fig. 4a and d, Supplementary Table 6). For example, while mRNA levels of *Ccr6* were decreased in Th1 and Th2 cells, the protein levels were almost unchanged. Similar to *Ccr6*, the expression of *Irf7* in Th17 and iTregs was decreased only at the mRNA level. Taken together, these findings suggest that T cell activation programs and cytokine-induced polarization act jointly to modulate Th cell subset-specific profiles at the transcript and protein level in addition to influencing the regulation of proteins uncorrelated with mRNA expression. To identify specific molecules that exhibit different expression patterns at the mRNA and protein levels, we first confirmed the mRNA and protein expression of subset-specific transcription factors using quantitative RT-PCR and flow cytometry analyses. As in multi-omics analyses, a FACS analysis showed that the levels of T-bet, GATA3, RORγt, and FOXP3 in each Th cell subset were significantly upregulated compared to Th0 cells at the protein level (Supplementary Fig. 3a and e). Furthermore, quantitative RT-PCR demonstrated the upregulation of the mRNA expression of these Th subset-specific genes (Supplementary Fig. 3f and i). We next focussed on specific immune-related genes such as *Irf7*, *Tnfsf8* [CD30L], *Pdcd1lg2* [PD-L2], and *Ccr6*, which showed uncorrelated expression profiles between the mRNA and protein levels (Fig. 4a and d). IRF7 is a key transcription factor in response to viral infections and CD4^+^ T cell in IRF7-deficient patients shows dysregulated IFNγ production.^32^ CD30L-deficient mice showed impaired Th1 responses against *M. bovis* bacillus Calmette-Guérin infection, suggesting that CD30L/CD30 signalling induced by CD30^+^-CD30L^+^ T cell interaction play an important role in amplification of Th1 responses.^33^ PD-L2 is known to inhibit aberrant T cell activation. Knockout of PD-L2 results in the excessed IFNγ production in Th1 cells.^34^ CCR6 is induced by TGFβ stimulation, which is important for the differentiation of Th17 and iTreg cells.^35^ Lack of CCR6 in Th17 cells reduces the severity of experimental autoimmune encephalomyelitis and Th17 and Treg recruitment into inflammatory tissues. We observed that the mRNA expression of *Irf7* was decreased to less than 10% in Th2, Th17, and iTregs cells compared to Th0 cells (Fig. 4e). However, similar to the results of multi-omics analyses, the protein expression of IRF7 was almost unchanged in each Th cell subset (Fig. 4f). Our combined analyses also showed that *Tnfsf8* [CD30L] showed greater changes in protein levels than in mRNA levels in each Th cell subset. Consistent with these results, we confirmed that the expression of protein CD30L on Th17 cells was much lower than on Th0 cells (Fig. 4g and h, Supplementary Fig. 3j) (Th17: 0.03%). In contrast, the mRNA levels of *Tnfsf8* [CD30L] in Th17 and iTregs were moderately decreased compared to that in Th0 cells (Fig. 4g and h) (Th17: 24.6%). We also observed that expression of PD-L2 protein was highly upregulated in Th2 cells, but upregulation of mRNA expression was not observed (Fig. 4i and j). In contrast, although we observed that downregulation of *Ccr6* mRNA in Th1 and Th2 cells compared to Th0 cells, protein expression of CCR6 was not significantly changed (Fig. 4k and l). In summary, our multi-omics analyses establish the proteogenomic profiles of T cell, delineating dynamic signalling networks in T cell differentiation and post-transcriptional regulation underlying T cell effector program.

## 4. Discussion

Although critical factors for the regulation of effector Th cell differentiation have been well studied, dynamic changes in the mRNA and protein levels during differentiation among effector Th cell subsets have been unclear. Using advanced molecular profiling technologies, we characterized changes in the proteogenomic profiles of effector Th cell subsets during differentiation. The goal of this study was to characterize Th cell differentiation at the molecular level, focussing on the modulation of in-depth proteogenomic profiles as the molecular phenotype of Th cells. In this regard, we consider the acquisition of the proteogenomic profile, where both the mRNA and protein profiles are simultaneously measured from the same sample, critical for depicting the cellular functional state: Elucidation of only the mRNA or protein profile is not sufficient for this purpose, as the proteins themselves take on the cellular function, whereas the mRNA level is a factor determining the cellular protein level. While previous study carried out a multi-omics comparative analysis of human naïve CD4^+^ T cells and memory CD4^+^ T cells from a single donor,^36^ we focussed on proteogenomic profiles of differentiation of murine naïve CD4^+^ T cells to five different effector Th cell subsets, including Th0, Th1, Th2, Th17, and regulatory T cells in this study. Effector Th cell subsets, including functionally distinct Th1, Th2, Th17, and Treg cells, are differentiated *via* the stimulation of TCR and cytokine to participate in host defense, allergic and autoimmune responses. In addition to analysis of these Th cell subsets, we also analysed nonpolarized Th0 cells induced by TCR stimulation alone as a control. This allows for a more accurate evaluation of the impact of cytokine signalling on the global changes of RNA and protein in activated T cells. Furthermore, because our multi-omics analyses have allowed us to measure mRNA and protein levels almost in a similar depth from the same sample, we intended to systematically generate proteogenomic datasets of Th cell differentiation and extract proteogenomic signatures reflecting the cellular dynamics of the Th cell differentiation processes in this study. These datasets are, to the best of our knowledge, the first of their kind for Th cell differentiation and are expected to provide us with a solid and indispensable basis to gain a comprehensive understanding of the mechanism underlying Th cell differentiation.

The proteogenomic data allowed us to confirm that the Th cell differentiation process could be divided into two steps: TCR activation- and cytokine-mediated cellular responses. TCR activation-mediated cellular responses were common in all Th cell subsets, but cytokine-mediated cellular responses determined the final destiny of each Th cell subset. We, therefore, obtained proteogenomic signatures of TCR activation and cytokine-induced differentiation to each Th cell subset for the first time. The obtained signatures contained many lines of information consistent with previous reports and further clarified many facts that might have some biological significance in Th cell differentiation.

TCR stimulation dramatically changes the expression profiles of mRNA and protein (UP: 2800, DOWN: 2201 in RNA-seq and UP: 1924, DOWN: 1683 in Proteome). Unexpectedly, among the differentially expressed genes, <10% were found to have an immune-related function. Regarding differentially expressed genes, excluding immune-related ones, molecules related to the cell cycle, cell division, and DNA replication were highly upregulated. We also observed that the number of differentially expressed genes that changed concordantly at both the mRNA and protein levels was less than half (UP: 1042, DOWN: 697). In other words, approximately half of differentially expressed genes exhibited discrepancies between changes at the mRNA and protein levels. In fact, there are many reports suggesting that TCR activation differentially affects translational rates and protein turnover rates of respective proteins, as described below.

Previous quantitative proteomic analyses showed that over 1,000 proteins undergo ubiquitination during TCR activation.^15^ This post-translational modification may be responsible for uncorrelated changes in the mRNA and protein expression caused by TCR stimulation.^7^ Furthermore, analyses of whole proteomes and phospho-proteomes indicate early dynamic phosphorylation events after 2 h of TCR stimulation and late amplification of both protein phosphorylation and expression, which collectively drive T cell activation.^14^ These data suggest that the Th cell subset fate decision during TCR activation is determined in the early phase. In addition to the importance of protein ubiquitination and phosphorylation events during TCR stimulation, it has been recognized that T cell switches their intracellular metabolic process to meet the energy requirements associated with their proliferation, activation, and specific functions.^2–5,37^ Although our GO analysis demonstrated the downregulation of genes related to the lipid metabolic process, we noted the upregulation of key enzymes relevant to lipid biosynthesis, including, *Acaca, Fasn, Fads2, Scd2, Hmgcr*, and *Hmgcs1*. We previously showed that PPARγ and SREBP1 directly control the expression of genes involved in the fatty acid uptake and synthesis programs.^38^ This regulation is required for the early activation and proliferation of CD4^+^ T cells.^38^ Ricciardi et al. also showed that the high glycolytic and fatty acid synthesis capabilities, as detected by mRNA levels in naïve CD4^+^ T cells, are not matched by protein expression. The translation of pre-accumulated mRNAs encoding *Acaca* regulates T cell metabolism.^39^ Upon TCR activation, the poised translational machinery is activated, resulting in the translation of ACC1 mRNAs and linking metabolism to effector cell fate.^39^

We also sought to identify the differences induced by cytokine stimulation during TCR activation. Th0, Th1, and Th2 cells show similar proteogenomic profiles to each other, and these similarities were captured by both transcriptomes and proteomes after 48 h of cell culture. In contrast, Th17 and iTregs were more similar to each other than to other cell states in PCA space. Consistent with our results, a previous multi-omics study also reported that, even after 16 h of cell culture, the transcriptome and proteome profiles of Th17 cells and iTregs were different from those of Th0, Th1, Th2, and type I interferon-treated Th1 cells.^29^ Because TGFβ is a cytokine commonly used to induce Th17 or Treg differentiation, we consider the differences between the two Th subsets as possibly resulting from TGFβ treatment. The TGFβ receptor-regulated adaptor molecules Smad2 and Smad3 as well as Smad-independent signalling are required to induce the expression of RORγt and FOXP3 in Th17 and Tregs, respectively.^30,31,40,41^ Smad2 and Smad3 are redundantly required for the regulation of TGFβ-mediated regulatory T cell generation.^40^ Naïve CD4^+^ T cells derived from Smad2/3 double-knockout mice failed to induce the expression of FOXP3 or exhibit a suppressive function.^30^ A previous microarray analysis showed that a large proportion of TGFβ-regulated genes were Smad2-/3-dependent. Gene deletion of Smad2, Smad3, or Smad2/3 suppressed 64% of TGFβ-induced genes. Those genes include *Foxp3*, *Ahr*, *Irf8*, and *Ikzf4*, and our proteogenomic analysis showed similar results. In contrast, TGFβ signalling is required for Th17 differentiation dependent on the JNK/Jun axis rather than independent of Smad2/3.^40^ In fact, genetic deletion of *Junb* inhibits differentiation of naïve CD4^+^ T cells to Th17 cells.^41^

The proteogenomic analysis of Th cell differentiation gave us a chance to reconsider the relationship between the mRNA and protein levels. The quantitative relationship between the mRNA and protein levels has already been discussed and mathematically modelled, even at a single-cell resolution,^6^ and these levels are determined by the balance between rates of synthesis and degradation. However, the mRNA level is a factor that controls the synthesis rate of protein.^42,43^ Thus, there is no pre-determined relationship between accumulated levels of mRNA and protein in principle. Rather, the relationship between the mRNA and protein levels may be another marker that sensitively reflects modulation of cellular states. However, we are inclined to believe that the protein level has a high correlation with the corresponding mRNA level in some way, as in-depth proteome measurements have been far less popular than non-targeted mRNA profiling. In this regard, because cellular differentiation is always accompanied by changes in cellular states, we should pay attention to the discrepancies between mRNA and protein profiles to ensure we have a comprehensive understanding of the cell differentiation processes on a molecular basis. The proteogenomic data reported in this study clearly show that the correlation between mRNA and protein levels varies widely among genes and among differentiation stages, implying the importance of the simultaneous acquisition of both mRNA and protein levels as a molecular phenotype of the biological system. Protein levels offer us crucial information directly relevant to cellular functions, while mRNA levels give important insights into how protein levels are modulated. We expect proteogenomic datasets to deepen our understanding of biological systems in general.

## Supplementary Material

dsac054_suppl_Supplementary_Figures

dsac054_suppl_Supplementary_Table_S1

dsac054_suppl_Supplementary_Table_S2

dsac054_suppl_Supplementary_Table_S3

dsac054_suppl_Supplementary_Table_S4

dsac054_suppl_Supplementary_Table_S5

dsac054_suppl_Supplementary_Table_S6

dsac054_suppl_Supplementary_Data

## Data Availability

The RNA-seq data have been deposited in the Gene Expression Omnibus at NCBI (https://0-www-ncbi-nlm-nih-gov.brum.beds.ac.uk/geo/) under accession number GSE210222. The MS files have been deposited to the ProteomeXchange Consortium via the jPOST partner repository^25^ (http://www.proteomexchange.org/) with the dataset identifier PXD036065.
