# Supplementary figures and images for: Characterization of proteogenomic signatures of differentiation of CD4+ T cell subsets
Source: DNA Res. 2022 Dec 29;30(1):dsac054. doi: 10.1093/dnares/dsac054 (PMC9886070; doi:10.1093/dnares/dsac054)

Supplementary Figure 1

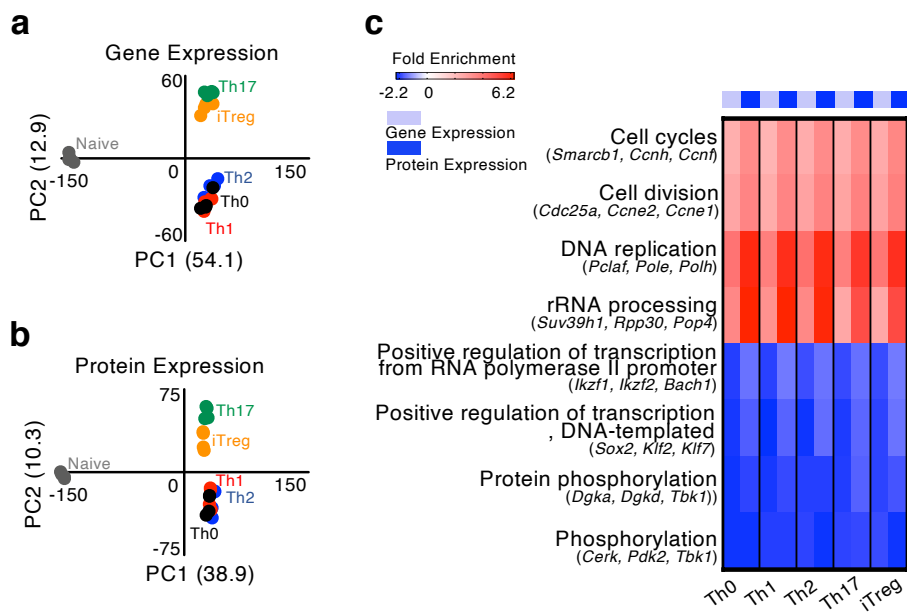

Supplementary Figure 2

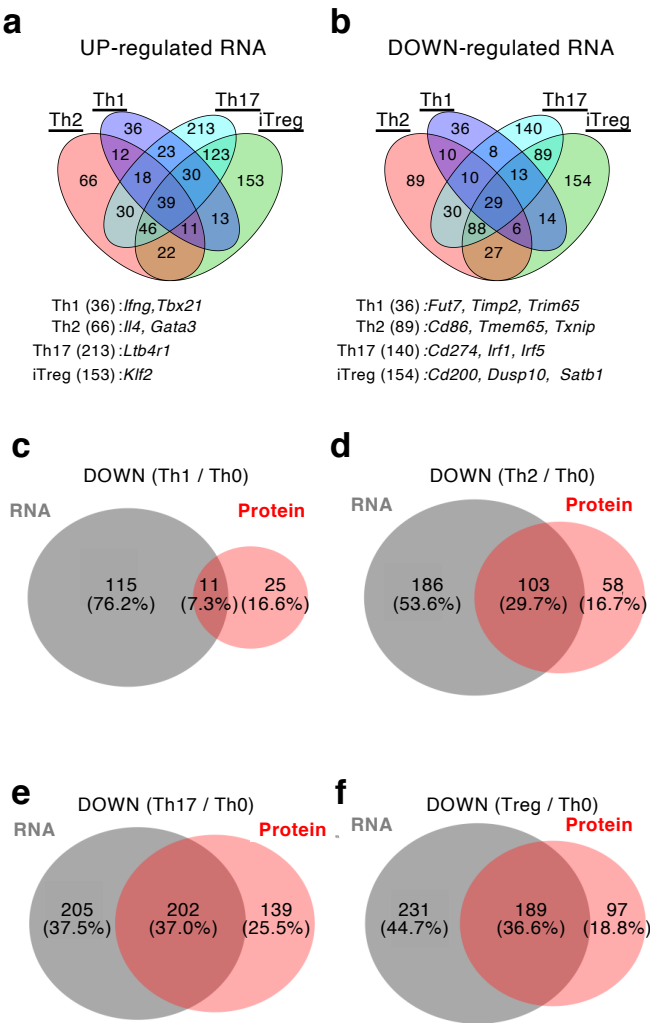

Supplementary Figure 3

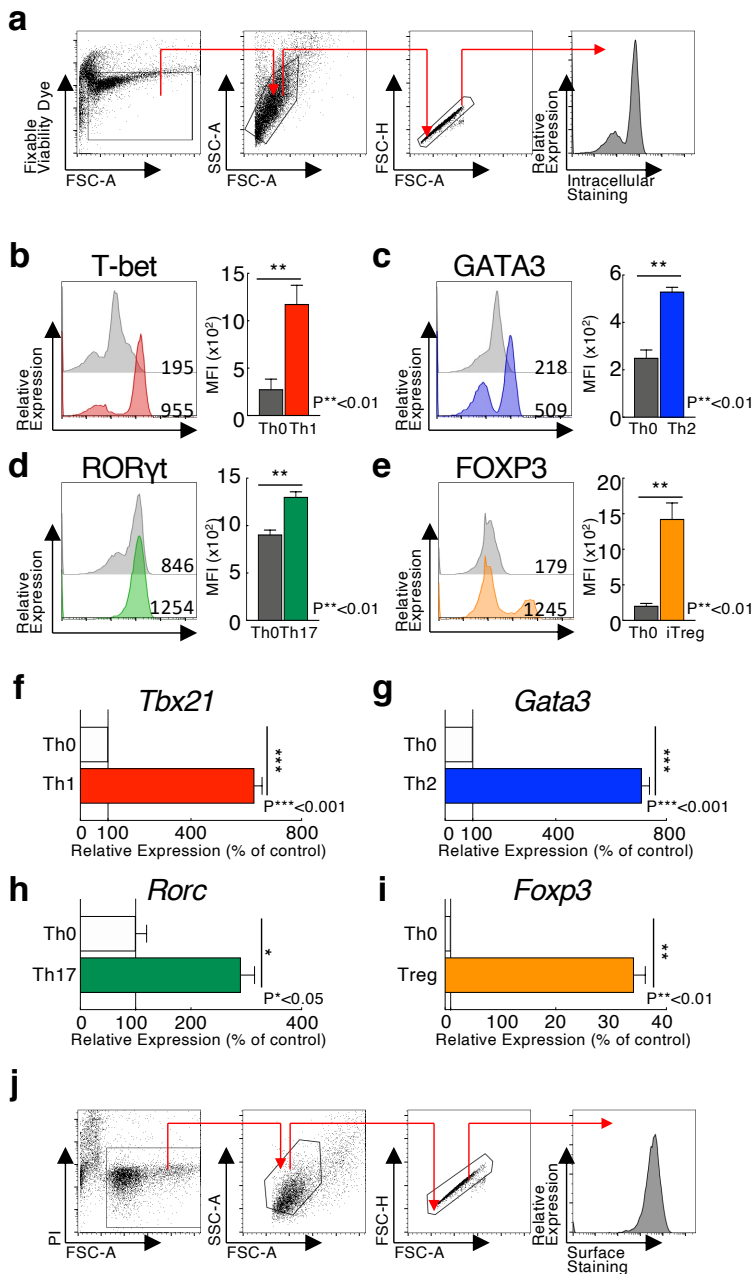

Supplement: dsac054_suppl_Supplementary_Figures [file dsac054_suppl_supplementary_figures.pdf]
