# Supplementary material for: Characterization of proteogenomic signatures of differentiation of CD4+ T cell subsets
Source: DNA Res. 2022 Dec 29;30(1):dsac054. doi: 10.1093/dnares/dsac054 (PMC9886070; doi:10.1093/dnares/dsac054)
Supplement: dsac054_suppl_Supplementary_Data [file dsac054_suppl_supplementary_data.docx]

**Legend**

**Supplementary Fig.1** TCR-mediated activation, rather than cytokine stimulation, provided the major impact on the proteogenomic profile related to Fig. 1.

**(a, b)** PCA plot of gene or protein expression profiles by RNA-sequencing (**a**) or proteome analysis (**b**), including naïve CD4, Th0, Th1, Th2, Th17 and iTreg cells. **(c)** Ontology analysis of RNA-sequencing and proteome analysis was performed in Th0, Th1, Th2, Th17 and iTreg cells as compared to naive CD4 T cells by using DAVID software (2.0-fold increase or decrease). FDR values are on −log 10 value.

**Supplementary Fig.2** Comparison of gene and protein expression among Th subsets related to Fig. 3.

**(a, b)** Venn plots of upregulated (**a**) or downregulated (**b**) genes analyzed by RNA-sequencing. Th1, Th2, Th17, and iTreg cells are compared against Th0 cells, respectively. (**c-f**) Venn diagram showed overlaps and differences between 2.0-fold decreased genes in Th1 (**c**), Th2 (**d**), Th17 (**e**), and iTreg (**f**) cells as compared to Th0 cells.

**Supplementary Fig.3** Confirmation of RNA-seq and LC-MS/MS results by FACS and quantitative RT-PCR analysis related to Fig. 4.

**(a)** Gating strategy of intracellular staining is shown. **(b-e)** Intracellular staining and flow cytometry analyzing of T-bet (**b**), GATA3 (**c**), RORγt (**d**) or FOXP3 (**e**) in Th1, Th2, Th17 or iTreg cells relative to Th0 cells. Mean fluorescence intensities (MFI) are shown. Summary data of three independent experiments of each protein expression are shown here. **(f-i)** qRT-PCR analyses of the relative expression of *Tbx21* (**f**), *Gata3* (**g**), *Rorc* (**h**), or *Foxp3* (**i**) in Th1, Th2, Th17 or iTreg cell compared to Th0 cells. Relative expression (normalized to *Hprt*) with SD is shown. **(j)** Gating strategy of surface staining is shown.
